# Supplementary material for: Relative tumor volume has prognostic relevance in canine sinonasal tumors treated with radiation therapy: A retrospective study
Source: PLoS One. 2022 May 27;17(5):e0269083. doi: 10.1371/journal.pone.0269083 (PMC9140277; doi:10.1371/journal.pone.0269083)
Supplement: S1 Table — GTVabs: Absolute tumor volume; GTVrel_W: Tumor volume relative to weight; GTVrel_BSA: Tumor volume relative to body surface area; GTVrel_NC: Tumor volume relative to nasal cavity; IQR: Interquartile range; SD: Standard deviation. (PDF) [file pone.0269083.s001.pdf]

S1 Table. Patient and tumor characteristics for part one (n=49).

**Breed**<br>n= (%)	Mixed breed: 12 (24.5%),<br>Golden Retriever: 4 (8.2%),<br>Labrador Retriever: 4 (8.2%),<br>Fox terrier: 2 (4.1%)<br>French Bulldog: 2 (4.1%),<br>Jack Russell Terrier: 2 (4.1%),<br>Beagle, Border Collie, Boxer, Cairn Terrier, Chihuahua, Cocker Spaniel, Collie, Dobermann Pinscher, Dobermann, Dutch Shepherd, Flat Coated Retriever, Irish Setter, Irish Wolfhound, Malinois, Mops, Perro de Agua Español, Podenco, Poteivin, Rottweiler, Shar-Pei, Weimaraner, Welsh Corgi, West Highland White Terrier*:*<br>1 (2.0 %) each
**Head conformance**<br>n= (%)<br>Brachycephalic<br>Meso-/ dolichocephalic	 <br>5 (10.2%)<br>44 (89.8%)
**Weight** (kg)<br>mean (±SD)	 23.6 (±13.0)
**BSA** (m^2)<br>mean (±SD)	 0.8 (±0.3)
**Nasal Cavity** (cm^3)<br>mean (±SD)	 107.4 (±54.6)
**GTVabs** (cm^3)<br>median [IQR]	 34.20 [15.35, 66.60]
**GTVrel_W**	

median [IQR]	1.71 [1.12, 2.25]
**GTVrel_BSA**	
median [IQR]	42.10 [27.02, 68.87]
**GTVrel_NC**	
median [IQR]	0.36 [0.26, 0.50]
**CTV** (cm^3)	
median [IQR]	75.30 [43.05, 145.10]
**PTV** (cm^3)	
median [IQR]	114.20 [63.65, 202.05]

GTVabs: absolute tumor volume; GTVrel_W: tumor volume relative to weight; GTVrel_BSA: tumor volume relative to body surface area; GTVrel_NC: tumor volume relative to nasal cavity; IQR: interquartile range; SD: standard deviation
