# Supplementary material for: Relative tumor volume has prognostic relevance in canine sinonasal tumors treated with radiation therapy: A retrospective study
Source: PLoS One. 2022 May 27;17(5):e0269083. doi: 10.1371/journal.pone.0269083 (PMC9140277; doi:10.1371/journal.pone.0269083)
Supplement: S2 Table — GTVrel_W: Tumor volume relative to weight; GTVrel_BSA: Tumor volume relative to body surface area; GTVrel_NC: Tumor volume relative to nasal cavity. (PDF) [file pone.0269083.s002.pdf]

S2 Table. Correlations between relative GTVs and dogs' sizes.

Correlations	GTVrel_W	GTVrel_BSA	GTVrel_NC
**Weight**			
r	0.002	0.21	0.06
P- value	0.99	0.14	0.70
**BSA**			
r	0.0035	0.22	0.04
P- value	0.98	0.135	0.79
**Nasal cavity**			
r	0.06	0.26	- 0.026
P- value	0.67	0.07	0.86

GTVrel_W: tumor volume relative to weight; GTVrel_BSA: tumor volume relative to body surface area; GTVrel_NC: tumor volume relative to nasal cavity.
