# Supplementary material for: Relative tumor volume has prognostic relevance in canine sinonasal tumors treated with radiation therapy: A retrospective study
Source: PLoS One. 2022 May 27;17(5):e0269083. doi: 10.1371/journal.pone.0269083 (PMC9140277; doi:10.1371/journal.pone.0269083)
Supplement: Attachment — Submitted filename: Response to Reviewers.docx [file pone.0269083.s003.docx]

**Manuscript ID PONE-D-21-40338**

Dear Journal Editorial Team,

Dear Reviewers,

first of all, we would like to thank you for your time and effort to revise our manuscript. Your constructive feedback helped us to make individual paper sections more precise and fluent. Thus, we have adjusted the manuscript according to the suggested changes and would like to resubmit it for further review. We sincerely hope that you approve of our changes.

**Reviewer 1's Comments to the Authors:**

General Comments:
This is a retrospective study in dogs with sinonasal tumor treated with 2 radiation protocols where the author wants to determine if the GTV either absolute or relative can be use for prognosis of outcome. Overall, the primary research question as in is relative tumor size a more informative prognostic indicator than absolute tumor size is valid. However, there is major limitations to the study in its current form to answer the research question. Some of the stated objectives of their study like pattern of failure does not articulate well in the paper and they do not make its relevance clear. Also although not stated in their objective, the emphasis placed on the comparison between radiation protocol is not warranted as it stands.

In general the study is hard to read as the wording is imprecise and does not flow. The primary outcome of time to progression is not appropriate since less than half of the dogs (23/49 (47%)) had documented imaging progression and only a bit more than 2/3 of dogs actually had imaging follow-up. The author did not define what to them is progression (i.e. imaging, clinical signs) and do not provide us with adequate follow-up information timing of CTs, recommended follow-up, which clinical signs they used to determine progression etc. Since the cohort of dogs were presented at a conference 15 months ago, I would assume that the data is mature and would highly suggest that the authors uses overall survival and/or progression-free survival as a primary outcome rather than time to progression that is more biased.

| **Comment of reviewer** | ***Response of authors*** | **New wording of the respective passages** |
| --- | --- | --- |
| General comment: Also although not stated in their objective, the emphasis placed on the comparison between radiation protocol is not warranted as it stands. | *Thank you for your comment. The aim of our study was to raise the question whether we should start to report and evaluate relative rather than only absolute tumor volume and its prognostic importance. Although we reported the time to progression for both protocols separately, it was not our intention to emphasize comparison of the protocols. Our goal was to state that both protocols had a similar outcome. This is why we subsequently evaluated the impact of stage, absolute and relative tumor volume on outcome in all dogs together (no matter with which protocol they were treated).* | Line 243: There was no statistically significant difference between protocols (p=0.45) and further evaluation regarding influence of GTVs and tumor stage was therefore performed with all dogs together. |
| General comment: The primary outcome of time to progression is not appropriate since less than half of the dogs (23/49 (47%)) had documented imaging progression and only a bit more than 2/3 of dogs actually had imaging follow-up. | *We split our study into two parts. In part one we assessed the different correlations (body weight, nasal cavity and so on) and used all 49 dogs. In part two we evaluated imaging based response and outcome only in the 34 dogs with available follow- up CTs (e.g. there was no available imaging follow-up in 15 dogs). The primary outcome of time to progression was therefore assessed only in 34 dogs and 23/34 (68%) of the dogs had documented imaging progression. This is more than half of the population evaluated for this part of the study and should therefore be adequate.*  *Furthermore, our study specifically looked at imaging and imaging based progression (and not survival). We would therefore like to keep our endpoint “time to progression”.* | - |
| General comment: The author did not define what to them is progression (i.e. imaging, clinical signs) and do not provide us with adequate follow-up information timing of CTs, recommended follow-up, which clinical signs they used to determine progression etc… | *Progression was defined in the general material and methods section in line 129 and in the statistics section in line 165. We added further explanatory comments as suggested.*  *Follow-up information (time point of CTs) is visualized in Figure 4. As suggested, we added a more detailed description in the manuscript.* | Line 155: Tumor progression was therefore defined as an increase in tumor size according to computed tomography as described by Nell et al. [2] (and not according to new/ recurrent clinical nasal signs).  Line 158: Re-check CTs were recommended every 6 months after the end of radiation therapy or in case of newly occurring nasal signs (but could vary due to owner preference).  Line 273: The number of re-check CTs performed was as follows: One re-check CT was performed in sixteen dogs, two in ten dogs, three in four dogs, four in three dogs and five in one dog. Twelve dogs were imaged at the recommended time points 6 and 12 months after radiation therapy (and if still alive every 6 months thereafter). Of those dogs re-imaged at the recommended time points, 2/12 showed no clinical nasal signs but (only) clear disease progression according to CT 12 and 18 months after radiation therapy, respectively. Ten dogs were imaged at the defined time points but also showed mild to moderate clinical nasal signs. Twenty- two dogs were imaged due to suspected nasal tumor recurrence at other time points than the recommended ones.  Line 394 (limitations): Because clinical response is subjective and clinical progressive disease is difficult to differentiate from chronic rhinitis, we decided to assess the response according to imaging only. However, the lack of uniform follow-up regarding time-points also does not allow for an accurate tumor response comparison. Time points of re-check CTs varied: while some were routinely planned 6 and 12 months after radiation therapy (and some detected early, symptom-free failures), others were performed due to recurrent clinical signs and/or suspected tumor relapse at various time points. |
| General comment: Since the cohort of dogs were presented at a conference 15 months ago, I would assume that the data is mature and would highly suggest that the authors uses overall survival and/or progression-free survival as a primary outcome rather than time to progression that is more biased. | *Data has indeed matured and our other manuscript has recently been accepted in the Journal of Veterinary Radiology and Ultrasound. This reference is now included in our manuscript (as it should have from the beginning). Because overall survival and progression-free survival have already been described in the previous study, because endpoints with survival have their inherent flaws and because emphasis of our study is on imaging and imaging progression (not survival), we decided to use time to progression as endpoint for our current manuscript.* | - |
| Item1 Title: the title does not report the study design nor does it report the treatment that the dogs received. It lacks clarity. Authors need to offer a new one. | *Opinions and scientific writing literature differ regarding titles. We hereby offer a new title as suggested.* | Line 1: Relative tumor volume has prognostic relevance in canine sinonasal tumors treated with radiation therapy: A retrospective study |
| Abstract. Sentences and words are awkward exemple line 39-41 I cannot really understand the meaning of the sentence. Please see Strobe conference abstract for details to be included in the abstract. | *Clarification provided as suggested.* | Line 38: In contrast to absolute GTV (p<0.001), the relative GTVs were not correlated with dogs’ body sizes. |
| Introduction Item2 There is no rational or background for their second objective-pattern of failure in the introduction. I would suggest to remove the second part or somehow address it into a cohesive study design and research question. | *The section about failure pattern was removed throughout the manuscript as suggested.* | - |
| For the GTV- Reference study 1 and 2 both uses PFS as primary outcome and 1 uses OS also as primary outcome. Both uses GTV as a dichotomous variable as less or more than the median GTV which was not performed in the current study. Reference 3 does not report GTV and do not evaluate GTV with outcome. Reference 4 did specify that outcome (they used progression free-interval and eliminated dogs that did not have a 3 months recheck CT as they could not reliably evaluate the outcome with only a 6 months follow-up CT) was not the objective of their study and they concluded that:” specifically,therewas a trend for all investigators in that smaller the baseline tumorvolume, the better the outcome; however, once a particular tumorvolume was reached, this effect was lost (Figure7A).” So possibly would have been found prognostic if GTV had been measured as a dichotomous variable. Reference 5 did use GTV relative to nasal size volume similar to the present study and not absolute GTV. They used overall survival as primary outcome. So this illustrate that your primary research question is a great one but you should make the reader aware of the discrepancy in the literature as it comes to GTV measurement and primary outcome. And then design your study to more adequately answer the question. | *The research articles were cited more precisely, this section was therefore rewritten as a whole.* | Line 50: Tumor volume remains poorly described as a prognostic factor in dogs treated with radiation therapy for sinonasal tumors. The few reports that described tumor volume and its association with outcome showed contradictory results. On the one hand, neither relative tumor regression (decrease in tumor volume in relation to nasal cavity), nor absolute tumor volume and percentage volume change at 1.5-3 months after radiation therapy were associated with overall survival or progression-free interval [1, 2]. On the other hand, both, tumor volume and degree of tumor response to radiation therapy were found prognostic for progression-free survival and overall survival [3]. In this latter study, initial tumor volume below the median and complete response yielded prolonged outcome. |
| Line 58-wording is strange what is an inspected factor? You want to introduce the idea of stage as it might correlate with your primary variable which is GTV. This is unclearly done. | *Adapted as suggested.* | Line 59: In contrast, tumor stage was evaluated in several studies. Nevertheless, the importance of tumor stage as prognostic parameter remains controversial. For example, some reports consider higher tumor stage to be prognostically unfavourable [4-6], but in other studies tumor stage was found not to be significantly associated with progression- free interval, progression-free survival or overall survival [2, 3, 7].  Line 75: Further insight into both, tumor volume and stage, their possible correlation and their influence on outcome are therefore warranted. |
| Line 68-69 not sure what this sentence means. Do you mean that since dogs have different body size/weight/nasal cavity measurement, you need to find a way to take this into account and modify the GTV measurement as needed and this is what you will do in this research project. | *Adapted as suggested.* | Line 69: Thus, tumor volume measurement seems to be important when evaluating response and outcome. However, dogs’ body sizes vary widely. A tumor of a certain volume might obstruct the nasal airway passage completely in a Chihuahua but occupies only a small part of the nasal cavity of a Great Dane. Thus, absolute tumor volume might not best represent the individual dog’s tumor size and its implications. Relative tumor volume might be better suited for different dog sizes and may shed further light on its ability to provide prognostic information. Further insight into both, tumor volume and stage, their possible correlation and their influence on outcome are therefore warranted. |
| Now you should state your hypothesis as it regards to GTV and leave the protocol/stage/pattern of failure for the material and methods. | *Thank you for your comment. There are different ways how to write an introduction. According to the courses we took and books we’ve read about scientific writing, one way is to give a small overview about the methodology of the manuscript. If it is important to you that we delete that part, we will certainly do so. Otherwise we would like to keep it in the manuscript as it is.* | - |
| I would remove the pattern of failure as one of your aim and hypothesis as it is a completely different unrelated research question. | *This was removed throughout the manuscript as suggested.* | - |
| Methods Item 4-Please specify study design-like single institution retrospective cohort study | *Corrected as suggested.* | Line 92: Datasets from a previous study were evaluated for this single institution retrospective cohort study [8]. |
| Item 5=Setting-please specify all the details of the study here as per Strobe. Location/dates/follow-up/data collection etc…. You cannot just refer all to a conference abstract it needs to be here in the paper | *The correct reference for the original study was added as suggested.*  *To avoid lengthy descriptions of information already described in detail in the original study, we added only few details as requested and referred to the original study for the remaining part. Please find all changes in the comments below. If you prefer to have a more detailed description in the current manuscript, please tell us what to add.* | - |
| Item 6-give how dogs were selected, how they were diagnosed , how dogs were allocated to the treatment protocol. Inclusion and exclusion criteria as to me you should only include dogs with histologically confirmed sinonasal tumor (maybe also the cytological one maybe) and exclude the benign tumors and possibly the zebra (i.e. nasal lymphoma/melanoma etccc) so you have a representative sample of typical sinonasal tumor that your can have external validity and with enough info that your reader can evaluate. | *Again, here we added more details as suggested but would like to refer to the original study for more detailed description as described above.*  *Please find our comment and our adaptations regarding histopathology in the comment below.* | Line 92: Datasets from a previous study were evaluated for this single institution retrospective cohort study [8]. The mentioned study was non-randomized and included dogs with non-lymphomatous, sinonasal tumors treated with either our regular radiation therapy protocol (10x4.2 Gy) or with a simultaneously- integrated boost protocol (SIB; GTV boosted to 10x4.83 Gy) and was approved by the Animal Ethics Council of the Canton of Zurich, Switzerland (permit number: ZH075/17). All subjects were client-owned dogs that underwent definitive-intent, daily (Monday-Friday) dynamic intensity-modulated radiation therapy (IMRT) at the Division of Radiation Oncology, Vetsuisse Faculty, University of Zurich.  Line 103: In part two of this study, we assessed response and outcome on a subset of irradiated dogs as described above and with available follow-up CTs. Because response and outcome were only evaluated based on imaging findings, inclusion criteria for part two were the same as for part one but required a re-check CT scan at any time point after the end of radiation therapy. |
| You should provide as much information on the histology of your cohort such as the sarcoma: how many were chondrosarcoma versus osteosarcoma versus fibrosarcoma, for the carcinoma-who was adenocarcinoma/squamous cell carcinoma versus undiffererentiated etc… And once again, the unknowns, benign and potentially others should not be included. | *Splitting into histopathological subtype would reveal very few dogs per group. As mentioned several times throughout the manuscript, we are aware of the flaws of our heterogeneous population. Please let us know if and where we can emphasize this further, if you wish us to do so.*  *We agree that inclusion of benign tumors or tumors with unknown histology could have influenced outcome. We would like to keep those dogs in the outcome analysis but added more explanatory details in the results, discussion and conclusion section.* | Line 190: In all three dogs with unknown tumor histology, a biopsy was attempted but was non-diagnostic. The dogs were included because CT characteristics were highly suspicious for a malignant nasal tumor (space-occupying soft tissue attenuating mass with aggressive bone lysis). The two dogs with benign tumors were included because they showed large tumor extension and complete obstruction of the nasal pathways incompatible with acceptable quality of life (euthanasia was considered by both owners). Histopathological diagnosis was an angiofibroma in one dog (local progression 220 days after the end of radiation therapy) and extensive polyp formation in the other dog (euthanized 500 days after the end of radiation therapy due to breathing difficulties).  Line 200: Table 1: Esthesioneuroblastoma  Line 203: *: angiofibroma, extensive polyps  Line 392 (limitations): Especially the decision to include two dogs with benign tumors and three with unknown histology could potentially have influenced outcome.  Line 405: We confirmed our hypothesis that *relative* rather than absolute GTV influenced TTP in our cohort of dogs. Also, larger tumor volume relative to nasal cavity was correlated with higher tumor stage. However, our study population was small and heterogeneous, our results therefore need to be interpreted with caution. Nevertheless, the results suggest that GTV *relative to nasal cavity* could serve as a prognostic factor for future studies. |
| Although the RT description is not the primary aim, you need to provide the details of dose prescription, GTV/CTV/PTV contouring. Who does the contouring etc….  Consider since there is no words limits to report everything required by Keyerleber MA, McEntee MC, Farrelly J, Podgorsak M. Completeness of reporting of radiation therapy planning, dose, and delivery in veterinary radiation oncology manuscripts from 2005 to 2010. Vet Radiol Ultrasound. 2012 Mar-Apr;53(2):221-30. | *The original study and the study from Keyerleber et al. were referenced as suggested. Even though there is no word limit, we would prefer not to repeat all planning aspects, as this has been done in depth in the original study. We added a chapter regarding treatment set-up, contouring and planning but in part refer to the original study. Please let us know if you prefer a more detailed description here.* | Line 116: Treatment set-up (rigid bite-block system and vacuum cushion), radiation equipment (Varian Clinac iX 6MV linear accelerator and ECLIPSE planning software version 10.0.28 or 15.1.25 (Varian Oncology Systems, Palo Alto, USA)), contouring of target volumes and organs at risk, and treatment planning was performed as described by Meier at al. [8]. In short, the GTV included the contrast enhancing mass seen in the co-registered contrast-enhanced CT images, as well as the lytic bone visualized in the bone window. The sinonasal cavity was included in the CTV, in case of GTV extension in that part or if it was filled with fluid. Additional cranio-caudal CTV extension in the sinonasal cavity or nasopharynx was 1.5 cm for carcinomas and benign tumors and 2 cm for sarcomas or unknown tumor histotypes. A 2 mm extension from the CTV led to the PTV. Contouring was performed by the responsible board-certified radiation oncologist (CRB or VM) as part of regular treatment.  Intended dose coverage was as follows: 98% of the PTV had to receive 95% or more of the prescribed dose of 42 Gy. For the SIB protocol, 98% of the GTV had to receive 95% or more of the prescribed SIB dose of 48.3 Gy. Dose maxima up to 107% of the respective dose levels in any volume and dose maxima of >110% in a small volume (<2%) within the PTV were allowed. Dose to target volumes as recommended by Rohrer Bley et al. and Keyerleber et al. [9, 10] was described in the original study [8].  The nasal cavity (NC) was defined according to an anatomical textbook and contoured retrospectively in all dogs starting rostrally from the nostrils to the ventral nasal meatus caudally, including the nasal septum and frontal sinus [11]. Contouring was performed by a veterinarian (FC) and checked by a board-certified radiation oncologist (VM). |
| Primary outcome needs to be well defined and ideally would be overall survival and/or progression-free survival. Please define how progression was determined if you will use progression-free survival as it can come from imaging but also clinical signs etc…. As it stands, less than 50% of your cohort had progression based on imaging. | *The endpoint was already described in the material and methods section (under the subtitle “Follow-up CT, response assessment and outcome” and “Statistical methods”). We added an additional sentence for clarification as suggested.*  *Different endpoints can be evaluated in cancer patients, all have their advantages and disadvantages. Overall survival has a very clear endpoint (the animal is either alive or dead, there is nothing unclear about that). However, this is not tumor-specific and is in part dependent on the owner (her or his tolerance of clinical signs before deciding for euthanasia), the age (tumor patients are often elderly and might not have a life expectancy of several years even without cancer) and concurrent illnesses. All this can influence overall survival. Progression-free survival (PFS) or time to progression (TTP) is more difficult to assess, especially in dogs with sinonasal tumors, as they might show intermittent nasal signs without tumor progression (chronic rhinitis) and clinical signs might start gradually over a longer period of time without the owner noticing. Progression-free survival is a conservative approach, as either death or progression counts as endpoint. Both PFS and TTP try to be more tumor-specific but are dependent on good follow-up and detection of new clinical signs or frequent imaging to detect imaging progression.*  *We chose TTP for this manuscript, because our endpoint was “progression according to CT”, which is not associated with survival. Moreover, overall survival and progression-free survival were already described in our recently accepted original study and we wanted to report a different endpoint.*  *TTP was based on imaging findings. This is why only the 34 dogs with available follow-up data were evaluated. Two thirds of this cohort (23/34 = 68%) showed imaging progression. This is more than half of the population evaluated for this part of the study.* | Line 155: Tumor progression was therefore defined as an increase in tumor size according to computed tomography as described by Nell et al. [2] (and not according to new/ recurrent clinical nasal signs). |
| Please define clearly how the initial GTV was contoured in which software and how it was defined. Was it contoured by only 1 rater. Mutliple rater, only once? Cannot refer to a conference abstract, details need to be in the current paper. | *The study we referred to has now been accepted and was correctly referenced. A description of contouring of the GTV and number of rater was added as suggested.*  *We agree that contouring of more than one rater would have been optimal. However, as stated throughout our manuscript, we are fully aware that our study has many limitations and that a larger study (with multiple raters) would be needed.* | Line 116: Treatment set-up (rigid bite-block system and vacuum cushion), radiation equipment (Varian Clinac iX 6MV linear accelerator and ECLIPSE planning software version 10.0.28 or 15.1.25 (Varian Oncology Systems, Palo Alto, USA)), contouring of target volumes and organs at risk, and treatment planning was performed as described by Meier at al. [8]. In short, the GTV included the contrast enhancing mass seen in the co-registered contrast-enhanced CT images, as well as the lytic bone visualized in the bone window.  Line 125: Contouring was performed by the responsible board-certified radiation oncologist (CRB or VM) as part of regular treatment. |
| Please clearly define the recommended follow-up in the methods and the follow-up achieved in the results. | *The recommended follow-up was added in the methods section. The follow-up executed was already visualized in Figure 4. More explanation was added in the results section.* | Line 158: Re-check CTs were recommended every 6 months after the end of radiation therapy or in case of newly occurring nasal signs (but could vary due to owner preference).  Line 273: The number of re-check CTs performed was as follows: One re-check CT was performed in sixteen dogs, two in ten dogs, three in four dogs, four in three dogs and five in one dog. Twelve dogs were imaged at the recommended time points 6 and 12 months after radiation therapy (and if still alive every 6 months thereafter). Of those dogs re-imaged at the recommended time points, 2/12 showed no clinical nasal signs but (only) clear disease progression according to CT 12 and 18 months after radiation therapy, respectively. Ten dogs were imaged at the defined time points but also showed mild to moderate clinical nasal signs. Twenty- two dogs were imaged due to suspected nasal tumor recurrence at other time points than the recommended ones. |
| Since I feel that the primary outcome needs to change and that the inclusion and exclusion criteria needs to be specified prior to even evaluate the results at this point. | *Please refer to our comment above with an explanation why we chose TTP as endpoint.*  *The inclusion criteria for part one and part two of the study are stated in the material and methods section. Please tell us more specifically what we can add to clarify this.* | - |

**Reviewer 2’s Comments to the Authors:**

General Comments:

The manuscript entitled "Do we need size correction for assessing tumor volume as a prognostic parameter in sinonasal tumors in dogs? " describes a study that looks at the effect of adjusted tumor size on outcomes. Overall this is interesting and while this study does not definitively answer the question proposed it does raise interesting points for further evaluation.

I have a few minor comments:

| **Comment of reviewer** | ***Response of authors*** | **New wording of the respective passages** |
| --- | --- | --- |
| Under the response and pattern of failure section (lines 253-268) it would be useful to the reader to present at least summary statistics regarding number of CT scans per dog evaluated, time to evaluation, how many were evaluated because of suspicion of progression or recurrence? | *More information regarding the number of CT scans and suspected progression was added as suggested.*  *The section about pattern of failure was removed as suggested by reviewer 1.* | Line 273: The number of re-check CTs performed was as follows: One re-check CT was performed in sixteen dogs, two in ten dogs, three in four dogs, four in three dogs and five in one dog. Twelve dogs were imaged at the recommended time points 6 and 12 months after radiation therapy (and if still alive every 6 months thereafter). Of those dogs re-imaged at the recommended time points, 2/12 showed no clinical nasal signs but (only) clear disease progression according to CT 12 and 18 months after radiation therapy, respectively. Ten dogs were imaged at the defined time points but also showed mild to moderate clinical nasal signs. Twenty- two dogs were imaged due to suspected nasal tumor recurrence at other time points than the recommended ones. |
| While the English overall is quite good there are multiple points where it is a bit awkward. For example line 282 "Herein, we revised" do you mean We evaluated? Perhaps consider having a native English language speaker review the manuscript. | *Thank you for your comment, we corrected this to the best of our ability.*  *At the same time - even if completely reasonable - we would like to emphasize that we are aware of not being native English language speakers. We are working continuously on improving our English and scientific language skills (books, courses).*  *Our manuscript was already proofread by a native English language speaker prior to submission, we are sorry if not all mistakes were corrected.* | Line 301: In the study presented herein, we evaluated tumor volume as a prognostic factor in a heterogeneous dog population with sinonasal tumors. |
