# Supplementary material for: Relative tumor volume has prognostic relevance in canine sinonasal tumors treated with radiation therapy: A retrospective study
Source: PLoS One. 2022 May 27;17(5):e0269083. doi: 10.1371/journal.pone.0269083 (PMC9140277; doi:10.1371/journal.pone.0269083)
Supplement: Attachment — Submitted filename: Review#2-Plos-One GTV paper.docx [file pone.0269083.s004.docx]

Review #2-Relative tumor volume has prognostic relevance in canine sinonasal tumors treated with radiation therapy: A retrospective study.

Good work on improving the manuscript. Some of my major concerns have not been addressed and I will leave it up to the editor to make a decision.

Overall, this is a cohort study of dog with sino-nasal tumor treated with RT-either 10 x 4.2 Gy or boost protocol 10 x 4.83 Gy. The purpose of the study was to look if GTV either absolute, relative to KG, BSA or nasal cavity was associated with stage and if these different GTV modification was prognostic using TTP as the primary outcome with progression based on follow-up imaging only.

Hypothesis: relative rather than absolute GTV influences time to progression (TTP) and that a larger tumor volume is correlated with a higher tumor stage.

Citation #8-Unfortunately, this paper is not available to the reviewer and would need to be in order to evaluate the current study but the current paper should stand on its own without having the read the citation 8 paper to evaluate the current study.

Inclusion criteria: Not really completely provided-histologically/cytologically diagnosed sino-nasal tumor? Would argue that benign histology and diagnosis based on imaging should not be included-if the authors wants to include, they have to provide rational here in the inclusion criteria. Reference to citation #8 for inclusion criteria in the study is not enough-all inclusion criteria needs to be specified in this current manuscript. Also need to provide the study period timelime.

Exclusion criteria: Also need to be specified. Did they exclude any dogs with sino-nasal tumor that were treated with the RT protocol during the study period? If so why? If not-need to be clear in the current paper. I personally think that the non-confirmed cases, the esthesioneuroblastoma and the benign histology should be excluded. If they are not excluded than it needs to specified in the abstract that cases with presumed nasal tumor, esthesioneuroblastoma and benign histology were included.

We start with 49 dogs with sino-nasal tumors (although based on the 34 dogs in part 2: 4 are presumed, 2 have benign histologies and 2 are esthesioneuroblastoma) treated with the RT protocol during the study period for part 1. Of those 15 dogs were excluded based on the fact that they don’t have follow-up CT to measure time to progression (TTP). However, if you use PFS or OS as primary outcome, you do not have to exclude these dogs. Selection bias is important here as 30% of the dogs with sino-nasal tumors treated during the study period are excluded from part 2. We also only have tumor characteristics in Table 1 of the dogs that were in the part 2 so we do not know the details of all the patients in the study.

TTP definition in the current form is “*TTP was evaluated by means of imaging and the three-dimensional response evaluation by Nell et al. (2020) and defined as the interval between radiation therapy start and disease progression according to the CT images [2]. “*

Going with this definition, the TTP could only be determined for 19 dogs (maybe more but this is based on figure 4 that I could see the GTV getting larger) so you have a TTP endpoint for only 55% (19/34) of dogs in part 2 and if you go with your overall population-you have a TTP endpoint of 38% (19/49) of dogs treated during the study period. This introduce information bias due to loss to follow-up that is very important.

As a generality, if you want to use TTP as an endpoint and surrogate endpoint for overall survival, you need to have follow-up imaging for all dogs at all specified timepoints. At least this is my understanding from the FDA document: <https://www.fda.gov/media/71195/download>.

*Dogs were censored if they died of a tumor-unrelated cause or if they were alive and free of progression at the time of data evaluation*.”

If you do not have follow-up imaging at the different timepoint, you cannot call them free of progression. You would need to censor at the last imaging timepoint without progression so my point is that you do not have as far as I can tell enough imaging to use TTP as a primary endpoint.

Here is what the FDA document says:

*“4. Time to Progression and Progression-Free Survival TTP and PFS have served as primary endpoints for drug approval. TTP is defined as the time from randomization until objective tumor progression; TTP does not include deaths. PFS is defined as the time from randomization until objective tumor progression or death, whichever occurs first.*

*The precise definition of tumor progression is important and should be carefully detailed in the protocol. Compared with TTP, PFS is the preferred regulatory endpoint. PFS includes deaths and thus can be a better correlate to overall survival. In TTP analysis, death events are censored, either at the time of death or at an earlier visit representing informative censoring (nonrandom pattern of loss from the study). PFS assumes that death events are randomly related to tumor progression. PFS can reflect tumor growth and be assessed before the determination of a survival benefit. Its determination is not confounded by subsequent therapy. For a given sample size, the magnitude of effect on PFS can be larger than the effect on overall survival. Data are usually insufficient to allow a robust evaluation of the correlation between effects on overall survival and PFS. Cancer trials are often small, and proven survival benefits of existing drugs are generally modest. Treatment effect measured by PFS can be a surrogate endpoint to support accelerated approval, a surrogate endpoint to support traditional approval, or it can represent direct clinical benefit based on the specific disease, context of use, magnitude of the effect, the disease setting, location of metastatic sites, available therapy, the risk-benefit relationship, and the clinical consequences of delaying or preventing progression in key disease sites (e.g., delay of new lesions in the brain or spine) or delaying administration of more toxic therapies. It is important to carefully define tumor progression criteria in the protocol. There are no standard regulatory criteria for defining progression. Applicants have used a variety of different criteria, including the RECIST criteria. The broad outline presented in most published PFS criteria should be supplemented with additional details in the protocol and statistical analysis plan (SAP). Because progression data can be collected from multiple sources (including physical exams at unscheduled visits and radiological scans of various types) and at different times, data collection for each assessment visit should be limited to a specified short time interval around the scheduled visit. Difficulties can arise in determining the event date and censoring date when data are collected over a prolonged time period. We recommend assigning the progression date to the earliest time when any progression is observed without prior missing assessments and censoring at the date when the last radiological assessment determined a lack of progression.*

So I cannot unfortunately support the use of TTP as defined in the current study form as the primary endpoint.
