# Supplementary material for: Relative tumor volume has prognostic relevance in canine sinonasal tumors treated with radiation therapy: A retrospective study
Source: PLoS One. 2022 May 27;17(5):e0269083. doi: 10.1371/journal.pone.0269083 (PMC9140277; doi:10.1371/journal.pone.0269083)
Supplement: Attachment — Submitted filename: Response to Reviewers.docx [file pone.0269083.s005.docx]

**Manuscript ID PONE-D-21-40338R1**

Dear Journal Editorial Team,

Dear Reviewers,

thank you for your time and effort to provide constructive feedback on our manuscript. We have reflected on the feedback and incorporated your suggestions into our manuscript. In our opinion, it has gained more clarity and quality as a result. We hereby resubmit it for further review and sincerely hope that you approve of our changes.

**Editor’s Comments to the Authors:**

| **Comment of editor** | ***Response of authors*** | **New wording of the respective passages** |
| --- | --- | --- |
| I agree with the opinion of Reviewer 1 that progression free interval / progression free survival is an inappropriate endpoint for this cohort of patients, given that not all patients were imaged and that the time of imaging varied. I agree that overall survival time is a more appropriate endpoint. Please adjust accordingly. | *Thank you for your comment. We do agree that time to progression (TTP) has its inherent flaws. We discussed the choice of endpoint again.*  Our manuscript is divided into two parts. In *part one*, we looked if the absolute tumor volume was correlated to the dogs’ weight or the nasal cavity size. The goal was to establish an appropriate relative tumor volume that considers the patients’ body size. All available dogs (n= 49) were therefore included in the first part, as we wanted to use as many tumor and dog sizes as possible. In *part two*, we aimed to investigate if this relative tumor volume may have an impact on outcome, e.g., time to progression.  Our current manuscript is based on data of a previous study, where outcome was reported as PFS and OS for the 49 dogs (“*Dose-escalated simultaneously integrated boost radiation protocol fails to result in a survival advantage for sinonasal tumors in dogs.*“). In the herein reported sub-population we include only the 34 dogs with available imaging follow-up, having our focus on tumor re-growth.  Hence, while we do agree that TTP has its inherent flaws, we still consider it adequate for our cohort of patients in this context and would like to keep it as it is.  We are aware of our study’s limitations as mentioned in the manuscript. | Line 405: However, our intention was to start a discussion about relative, rather than absolute GTV as a possible prognostic factor in the future; further research is certainly needed.  Line 416: Therefore, given that not all patients were imaged and that the time of imaging varied, our results need to be interpreted with caution.  Line 425: While our study population was small and heterogeneous, we want to stimulate the idea, that GTV *relative to nasal cavity* should be evaluated as a prognostic factor in future studies. |

**Reviewer 1's Comments to the Authors:**

General comments: Good work on improving the manuscript. Some of my major concerns have not been addressed and I will leave it up to the editor to make a decision.

Overall, this is a cohort study of dog with sino-nasal tumor treated with RT-either 10 x 4.2 Gy or boost protocol 10 x 4.83 Gy. The purpose of the study was to look if GTV either absolute, relative to KG, BSA or nasal cavity was associated with stage and if these different GTV modification was prognostic using TTP as the primary outcome with progression based on follow-up imaging only.

Hypothesis: relative rather than absolute GTV influences time to progression (TTP) and that a larger tumor volume is correlated with a higher tumor stage.

| **Comment of reviewer** | ***Response of authors*** | **New wording of the respective passages** |
| --- | --- | --- |
| Citation #8-Unfortunately, this paper is not available to the reviewer and would need to be in order to evaluate the current study but the current paper should stand on its own without having the read the citation 8 paper to evaluate the current study. | *Thank you for your comment. The manuscript we referred to was accepted several weeks ago. However, we had not received a DOI number and had to ask the journal repeatedly to provide it. As soon as the DOI number was available, we sent it to the VRU and asked it to be sent to you. We apologize if you have not received it.*  *The study we referred to has now been officially published (doi:10.1111/vru.13086). To avoid lengthy descriptions of information already described in detail in the original study, we added only few – but most important – details and referred to the original study for the remaining part.* | 8. Meier V, Staudinger C, Körner M, Bley CR. Dose-escalated simultaneously integrated boost radiation protocol fails to result in a survival advantage for sinonasal tumors in dogs. Vet Radiol Ultrasound. 2022. doi: 10.1111/vru.13086 |
| Inclusion criteria: Not really completely provided-histologically/cytologically diagnosed sino-nasal tumor? Would argue that benign histology and diagnosis based on imaging should not be included-if the authors wants to include, they have to provide rational here in the inclusion criteria.  Reference to citation #8 for inclusion criteria in the study is not enough-all inclusion criteria needs to be specified in this current manuscript. | *All inclusion criteria are now specified in the current manuscript as suggested.* | Line 103: Inclusion criteria for part one was a dog with a cytologically or histologically diagnosed sinonasal tumor. Dogs with unknown tumor histology or benign tumors were also included, either because of highly suspicious CT characteristics for a malignant nasal tumor or because of severe clinical nasal signs incompatible with acceptable quality of life. Not only including malignant sinonasal tumors seemed reasonable, as the goal of part one of the study was correlation of different tumor volumes to different dog sizes. |
| Also need to provide the study period timelime. | *Added as suggested.* | Line 93: The mentioned study was non-randomized and included dogs between June 2014 and May 2020 with non-lymphomatous, sinonasal tumors treated with either our regular radiation therapy protocol (10x4.2 Gy) or with a simultaneously- integrated boost protocol (SIB; GTV boosted to 10x4.83 Gy) and was approved by the Animal Ethics Council of the Canton of Zurich, Switzerland (permit number: ZH075/17). |
| Exclusion criteria: Also need to be specified. Did they exclude any dogs with sino-nasal tumor that were treated with the RT protocol during the study period? If so why? If not-need to be clear in the current paper. | *We added an explanatory comment as suggested.*  *In our previous study [8], all dogs with sinonasal tumors treated with either a regular or a boost 10-fraction protocol were included, i.e. none were excluded. Our current study was split into two parts. In part one we assessed the different correlations (body weight, nasal cavity and so on) and used all 49 dogs. In part two we evaluated outcome only in the 34 dogs with available follow- up CTs. That means we excluded 15 dogs for part two, because they didn’t have follow- up CTs. This is declared in the Materials and Methods section (Line 110-114) and the Results section (Line 197-199)* | Line 114: This means, cases were excluded for part two, if they didn’t have a follow- up CT. |
| I personally think that the non-confirmed cases, the esthesioneuroblastoma and the benign histology should be excluded. If they are not excluded than it needs to specified in the abstract that cases with presumed nasal tumor, esthesioneuroblastoma and benign histology were included. | *We elected to add this information in the abstract as suggested.* | Line 33: Here, also presumed sinonasal tumors, esthesioneuroblastomas and histologically benign tumors were included. |
| We start with 49 dogs with sino-nasal tumors (although based on the 34 dogs in part 2: 4 are presumed, 2 have benign histologies and 2 are esthesioneuroblastoma) treated with the RT protocol during the study period for part 1. Of those 15 dogs were excluded based on the fact that they don’t have follow-up CT to measure time to progression (TTP). However, if you use PFS or OS as primary outcome, you do not have to exclude these dogs. Selection bias is important here as 30% of the dogs with sino-nasal tumors treated during the study period are excluded from part 2. | *Thank you for your comment. The 49 dogs in the first part of the study were included because we wanted as many tumor and dog sizes as possible, in order to correlate tumor volume to different dog sizes.*  *Outcome (PFS and OS) in those 49 dogs was already reported in the previous study [8]. We specifically chose TTP to assess how long radiation therapy can control the tumors. The latter is not clear when OS or PFS are evaluated. Especially in dogs with sinonasal tumors, some owners develop a high tolerance towards epistaxis or reduced quality of life and might decide to put the pet to sleep rather late. This is most likely shown by the difference in OS and PFS or TTP in previous studies. To evaluate TTP objectively, we chose to include only the 34 dogs with available imaging follow-ups.The 34 patients of part two are therefore considered as single group. No further exclusion criteria were applied within this group.* | - |
| We also only have tumor characteristics in Table 1 of the dogs that were in the part 2 so we do not know the details of all the patients in the study. | *Tumor characteristics of all 49 dogs was already published previously (doi:10.1111/vru.13086). From our point of view, tumor types are not necessary for part one in the current manuscript (as outcome was only reported for 34 dogs).* | - |
| TTP definition in the current form is “*TTP was evaluated by means of imaging and the three-dimensional response evaluation by Nell et al. (2020) and defined as the interval between radiation therapy start and disease progression according to the CT images [2]. “*  Going with this definition, the TTP could only be determined for 19 dogs (maybe more but this is based on figure 4 that I could see the GTV getting larger) so you have a TTP endpoint for only 55% (19/34) of dogs in part 2 and if you go with your overall population-you have a TTP endpoint of 38% (19/49) of dogs treated during the study period. This introduce information bias due to loss to follow-up that is very important. | *Thank you for your comment and careful reading. In Figure 4, a few (3 dogs) were not coded as progressive by mistake. We apologize for this error and corrected Figure 4.*  *While our goal was to have imgaging of all dogs that showed clinical signs of progressive disease, some owners declined imaging due to the required anesthesia and costs. This is why dogs were considered to have tumor progression either due to progression on imaging or clear clinical signs. We are aware that it would have been better to have imaging proof of all patients. However, this is rarely the case in veterinary patients.*  *The primary outcome of time to progression was therefore assessed in 34 dogs and 23/34 (68%) of the dogs had documented imaging progression.*  *Regarding further comments about TTP please refer to our comments above and below.* | Figure 4  Line 183: Tumor progression was evaluated by means of imaging and the three-dimensional response evaluation by Nell et al. (2020). If no imaging was available but the dog showed clinical nasal signs compatible with progressive disease, the dog was considered to show progressive disease as well. TTP was defined as the interval between radiation therapy start and disease progression according to the CT images [2] or clear clinical nasal signs. |
| As a generality, if you want to use TTP as an endpoint and surrogate endpoint for overall survival, you need to have follow-up imaging for all dogs at all specified timepoints. At least this is my understanding from the FDA document: <https://www.fda.gov/media/71195/download>. | *We completely agree that imaging at the specified time points would have been needed. However, this was not the case.*  *In clinical veterinary oncology trials it is very difficult to implement the standardized recommendations from human medicine (as represented by the FDA document). While it is already challenging to obtain a large number of study patients, it is even more difficult to establish regular follow-up imaging. The most limiting factors for our clients are the costs (which are not covered by health insurances) and the required anesthesia (which always carries a certain risk for complications).*  *In addition – and as described above – our study was not intended to be a convincing outcome study. While our study points towards the finding that a correlation between relative tumor volume and outcome may exist, we are well aware that more research is needed. We mentioned several times in the manuscript, that our results need to be interpreted with caution. Our intention is to start the discussion about the relative tumor volume being of possible prognostic value.* | Line 405: However, our intention was to start a discussion about relative, rather than absolute GTV as a possible prognostic factor in the future; further research is certainly needed.  Line 416: Therefore, given that not all patients were imaged and that the time of imaging varied, our results need to be interpreted with caution.  Line 425: While our study population was small and heterogeneous, we want to stimulate the idea, that GTV *relative to nasal cavity* should be evaluated as a prognostic factor in future studies. |
| ”*Dogs were censored if they died of a tumor-unrelated cause or if they were alive and free of progression at the time of data evaluation*.”  If you do not have follow-up imaging at the different timepoint, you cannot call them free of progression. You would need to censor at the last imaging timepoint without progression so my point is that you do not have as far as I can tell enough imaging to use TTP as a primary endpoint.  Here is what the FDA document says:  *“4. Time to Progression and Progression-Free Survival TTP and PFS have served as primary endpoints for drug approval. TTP is defined as the time from randomization until objective tumor progression; TTP does not include deaths. PFS is defined as the time from randomization until objective tumor progression or death, whichever occurs first.*  *The precise definition of tumor progression is important and should be carefully detailed in the protocol. Compared with TTP, PFS is the preferred regulatory endpoint. PFS includes deaths and thus can be a better correlate to overall survival. In TTP analysis, death events are censored, either at the time of death or at an earlier visit representing informative censoring (nonrandom pattern of loss from the study). PFS assumes that death events are randomly related to tumor progression. PFS can reflect tumor growth and be assessed before the determination of a survival benefit. Its determination is not confounded by subsequent therapy. For a given sample size, the magnitude of effect on PFS can be larger than the effect on overall survival. Data are usually insufficient to allow a robust evaluation of the correlation between effects on overall survival and PFS. Cancer trials are often small, and proven survival benefits of existing drugs are generally modest. Treatment effect measured by PFS can be a surrogate endpoint to support accelerated approval, a surrogate endpoint to support traditional approval, or it can represent direct clinical benefit based on the specific disease, context of use, magnitude of the effect, the disease setting, location of metastatic sites, available therapy, the risk-benefit relationship, and the clinical consequences of delaying or preventing progression in key disease sites (e.g., delay of new lesions in the brain or spine) or delaying administration of more toxic therapies. It is important to carefully define tumor progression criteria in the protocol. There are no standard regulatory criteria for defining progression. Applicants have used a variety of different criteria, including the RECIST criteria. The broad outline presented in most published PFS criteria should be supplemented with additional details in the protocol and statistical analysis plan (SAP). Because progression data can be collected from multiple sources (including physical exams at unscheduled visits and radiological scans of various types) and at different times, data collection for each assessment visit should be limited to a specified short time interval around the scheduled visit. Difficulties can arise in determining the event date and censoring date when data are collected over a prolonged time period. We recommend assigning the progression date to the earliest time when any progression is observed without prior missing assessments and censoring at the date when the last radiological assessment determined a lack of progression.*  So I cannot unfortunately support the use of TTP as defined in the current study form as the primary endpoint. | *Thank you for your comment. The FDA document describes standardized recommendations for studies in human medicine. In clinical veterinary trials it is very difficult to fully implement these recommendations. The most limiting factors are the costs and anesthesia. Both make it difficult to have clients come back for imaging re-checks multiple times and even more difficult if the dog exhibits clear signs of local progression (visible mass on the back of the nose or exophthalmos). While we agree that in an ideal world, we would have imaging at the defined time points and at the start of recurrent clinical signs, this is probably not very realistic.*  *We realized that our definition of TTP was lacking in detail and adjusted it accordingly.*  *While PFS is a better correlate to overall survival, it is a conservative endpoint as both, progression and death are events. Because both overall survival and PFS were already reported in our previous study, we wanted to have a look at the data from a different angle. TTP (or PFI) only looks at the time until the tumor progresses. It is therefore an endpoint that does not say anything about survival but is able to give an idea on how long a tumor is well controlled after treatment. In a tumor that might allow the patient to survive several weeks to a few months after progressive disease was diagnosed (as demonstrated by the difference between survival and TTP/ PFS in many studies about sinonasal tumors in dogs), survival is a good and very clear endpoint but it does not tell us how long the treatment was able to stop or control the disease.*  *We do agree that TTP has its inherent flaws and we therefore mentioned several times in the manuscript that our results need to be interpreted with caution and that further research is needed to evaluate the prognostic significance of the relative tumor volume. We added additional comments to emphasize this.*  *In our opinion, the outlined limitations of our work should provide new suggestions for future work. We are fully aware that further research is needed.* | Line 183: Tumor progression was evaluated by means of imaging and the three-dimensional response evaluation by Nell et al. (2020). If no imaging was available but the dog showed clinical nasal signs compatible with progressive disease, the dog was considered to show progressive disease as well. TTP was defined as the interval between radiation therapy start and disease progression according to the CT images [2] or clear clinical nasal signs. Dogs were censored if they died of a tumor-unrelated cause or if they were alive and free of progression at the time of data evaluation.  Line 405: However, our intention was to start a discussion about relative, rather than absolute GTV as a possible prognostic factor in the future; further research is certainly needed.  Line 416: Therefore, given that not all patients were imaged and that the time of imaging varied, our results need to be interpreted with caution.  Line 425: While our study population was small and heterogeneous, we want to stimulate the idea, that GTV *relative to nasal cavity* should be evaluated as a prognostic factor in future studies. |

**Reviewer 2’s Comments to the Authors:**

General Comments:

Thank you for the response to my comments and I feel that the authors have addressed my concerns.

A few small suggestions:

| **Comment of reviewer** | ***Response of authors*** | **New wording of the respective passages** |
| --- | --- | --- |
| Line 137 change 10,1 to 10.1 | *Corrected as suggested.* | Line 143: The BSA was computed using the formula according to Pereira et al.: BSA (cm²) = 10.1 x weight (grams)^2/3^ [12]. |
| I am surprised that age and weight were normally distributed as this is often not the case. This is suggested since they are presented as means and methods state normally distributed data is presented this way. Would suggest confirming this. | *Added as suggested.* | Line 211: Table 1: *  Line 214: *: data was normally distributed according to Shapiro-Wilk test and is therefore presented as mean (±SD) |
| Line 168 - you state data for medians are presented as "median (95% confidence interval (95% CI) or range)" in tables you present with IQR - this is fine in my opinion but please add this to info in statistical section. | *Added as suggested.* | Line 175: Descriptive statistics were reported with mean (standard deviation) for normally distributed data and median (95% confidence interval (95% CI) or interquartile range (IQR)) for non-normally distributed data. |
